# Prevalence of human enteroviruses among apparently healthy nursery school children in Accra

**DOI:** 10.11604/pamj.2014.18.66.3232

**Published:** 2014-05-19

**Authors:** Juliana Attoh, Evangeline Obodai, Theophilus Adiku, John Kofi Odoom

**Affiliations:** 1 Department Of Microbiology, University of Ghana Medical School, Legon, Ghana; 2 Ghana Health Service, Ministry of Health, Ghana; 3 Department of Virology, Noguchi Memorial Institute for Medical Research, University of Ghana, Legon, Ghana

**Keywords:** Non-polio enterovirus, apparently healthy, school children, Accra

## Abstract

**Introduction:**

Human enteroviruses are common in children causing asymptomatic infections ranging from mild to severe illnesses. In Ghana, information on the prevalence of non-polio enterovirus causing acute flaccid paralysis is available but data on surveillance of these viruses in school children is scanty. Here, the prevalence of human enteroviruses among apparently healthy children in selected school in Accra was studied.

**Methods:**

Stool samples from 273 apparently healthy children less than eight years of age in 9 selected nursery schools were collected between December 2010 and March 2011and processed for human enteroviruses on L20B, RD and Hep-2 cell lines. Positive Isolates were characterized by microneutralisation assay with antisera pools from RIVM, the Netherlands according to standard methods recommended by WHO.

**Results:**

Of the 273 samples processed, 66 (24.2%) non-polio enteroviruses were isolated. More growth was seen on Hep-2C (46%) only than RD (18%) only and on both cell lines (34%). No growth was seen on L20B even after blind passage. Excretion of non-polio enteroviruses was found in all the schools with majority in BD school. Serotyping of the isolates yielded predominantly Coxsackie B viruses followed by echoviruses 13 and 7. More than half of the isolates could not be typed by the antisera pools.

**Conclusion:**

The study detected 13 different serotypes of non-polio enteroviruses in circulation but no poliovirus was found. BD school was found to have the highest prevalence of NPEV. Complete identification through molecular methods is essential to establish the full range of NPEVs in circulation in these schools.

## Introduction

Human enterovirus (HEV) belongs to the family Picornaviridae. They are among the most common human viruses, infecting an estimated number of a billion people annually worldwide [1]. Over one hundred immunologically distinct serotypes are known to cause infections in humans [2]. Even though the majority of infections are often asymptomatic and goes unnoticed, these viruses are also associated with occasional outbreaks [3, 4]. HEV are believed to spread mainly by fecal-oral routes which spread mostly within families. It is usually isolated in the highest titer and longest time in stool specimens but can also be isolated from respiratory secretions. Its transmission is usually between siblings, and an increased risk of virus spread is within crowded living accommodation [5]. People of all ages are at risk of manifesting symptoms of HEVs. Children have a higher rate of infection because of exposure, hygiene, and immunity status [5, 6].

Human enteroviruses are considered by many to be unimportant as human pathogens, however, they have been found to be associated with serious or even fatal diseases [7] (Kearney, 2001). Notable among these are polioviruses known to cause paralytic poliomyelitis. Other non-polio enteroviruses (NPEVs) have been shown to cause acute flaccid paralysis which mimics polio including Echoviruses which has been implicated in multiple human disease syndromes [8]. Since the last wild poliovirus was detected in Ghana in 2008, surveillance for poliovirus by acute flaccid paralysis has detected no wild poliovirus. To ensure that wild poliovirus transmission has been completely interrupted, surveillance for HEV among apparently healthy children in the population to supplement AFP surveillance is essential. This helps to identify gaps where Poliovirus transmission could occur undetected and allow the timely detection of an outbreak in a previously polio-free area [9]. Apparently healthy preschool-aged children are known to be the major reservoir for enteroviruses in the community [10]. The oral poliovaccine used for immunization, seeds the guts of children by inducing both circulating and secretory antibodies that protects against polio infection. However, various studies have shown that wild polio virus can circulate in a well-vaccinated population with or without clinical cases [11]. This has been exemplified by recent outbreaks of poliomyelitis in some parts of Africa which suggested that wild poliovirus can replicate asymptomatically in well-immunized persons and eventually infect susceptible individuals [12], as there is a continuous chain of person-to-person transmission [13]. Our study is aimed at determining the prevalence of human enteroviruses among apparently healthy school children less than 8 years of age in the Ablekuma South community.

## Methods

### Study area

Nine pre-schools in Ablekuma South Sub-Metro of the Accra Metropolitan Assembly were randomly selected. These include AME Zion (AME), Bishop Dally (BD), Dansoman Community (DC), Hijaz Islamic (HI), Korle-Gonno Girls (KG), Kitson Mills (KM), Mamprobi South 4 (MS), Matyrs of Uganda (MU) and Tunga Com Islamic (TI) kindergarten schools. The study was carried out during the dry (December-March) season of 2011. The area was selected on the basis of overcrowding and poor sanitation. Ablekuma South is a fishing community with low lying slums. It is a low socioeconomic area with low standard of living. Cluster of public schools and nurseries abound in the area and children attend school at later age.

**Study population** Children five years and below are expected to be in the nursery however, some children in public nurseries schools were usually above five years. Two hundred and seventy three children were enrolled in this study. Samples were collected from these children between December 2010 and March 2011. The children were apparently healthy and majority of them have received the complete vaccination for routine polio immunization as confessed by the mothers during their informed consent for selection. In addition, they had received supplemental vaccination during the national immunization day in March 2010. Total number of OPV was however unknown since doses during NIDs were not recorded and the vaccination cards were not presented. Stool specimens were collected from each participant with demographic data including age, sex, location and immunization history. All samples were transported at 4°C to the polio laboratory at the Department of Virology, Noguchi Memorial Institute for Medical Research (NMIMR), University of Ghana, Accra, for storage at -20°C until ready to be processed.

### Virus isolation and typing

Stool samples received at the World Health Organization (WHO) accredited Regional Polio Laboratory, NMIMR, were processed according to WHO Polio laboratory manual [14] (WHO,2004). Briefly, 4-8grams of specimen was treated in PBS with 10% chloroform and inoculated on Rhabdomyosarcoma (RD), Hep-2C human cell lines and L20B cell line derived from mouse to express the poliovirus receptor CD155. Both monolayer cell lines were seeded in culture tubes with 10% fetal calf serum (FCS) and changed to maintenance medium prior to inoculation. Cultures were incubated at 37°C and observed daily for cythopatic effect (CPE). Positive cultures were harvested and stored at -20°C while negative cultures observed for five days and re-passaged onto a new monolayer.

All positive cultures on RD cells were selected for serotyping using pools of antisera containing horse typing antisera against HEV serotypes prepared by the National Institute of Public Health and the Environment (RIVM), Netherlands. The typing was done according to the WHO polio laboratory manual [14]. Briefly, equal amount of 1:10 dilution of the isolate was mixed with different combinations of the antisera pools and incubated for 1 hr at 36°C before the addition of RD cells. Characteristics CPE was observed daily for five days. Antisera combination that prevented the formation of CPE identifies the virus [14]. All isolates that were not neutralized by the pools of antisera were subjected to microneutralization assay using antiserum specific for enterovirus 71 (E71).

### Data Analysis

Data analysis was done using SPSS version 19. The analysis involves frequency distribution of responses and cross tabulation of variables. The analyzed information was presented using tables, graphs, charts and other diagrams that depicted the pattern of findings.

### Ethical Issues

Ethical approval was obtained from the Ethical Committee of the College of Health Sciences, and Research Committee of the University of Ghana Medical School (UGMS). Informed consent was sought from guardian of subjects before the commencement of the study. All ethical considerations were adhered to. Data collected from the study was handled anonymously and confidentially. Samples had only the identification numbers of the subjects to ensure anonymity. We protected the confidentiality of patients through use of codes.

## Results

### Demographic characteristics

Stool samples were collected from two hundred and seventy three apparently healthy children under the age of 8 years to assess the prevalence of human enteroviruses in the Ablekuma south sub metro of the Accra Metropolitan Assembly. A total of 143/273 (52%) of subjects were female and the mean age of the study children was 4.6. The total OPV doses received by these apparently healthy children was unknown however in addition to the routine OPV doses received, all the children received their vaccination during the national immunization day on March, 2010, eight to nine months before stool specimen were collected.

### Serological characterization of NPEVs

**Figure 1:**
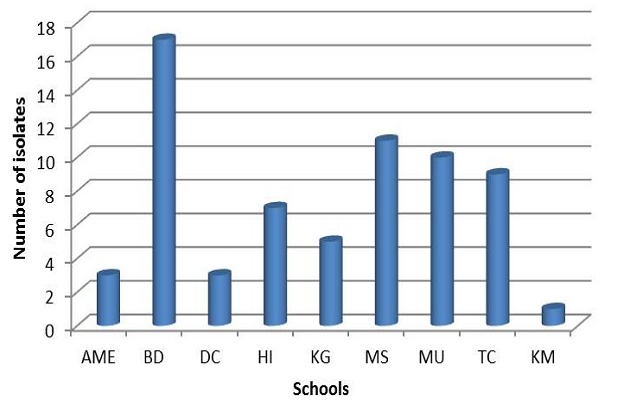
Distribution of non-polio enterovirus by school

**Figure 2:**
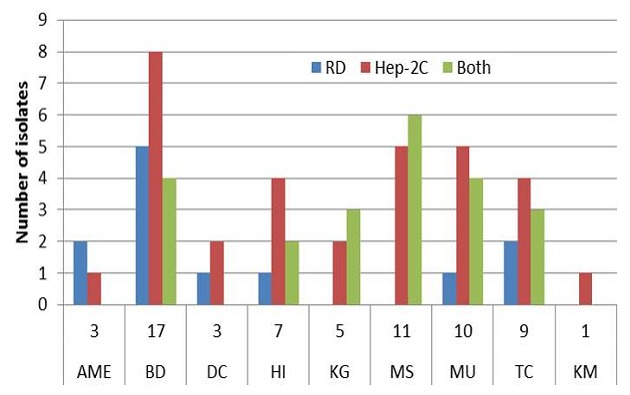
Number of isolates positive on different RD and Hep-2C cell lines by school

Stool suspensions from 273 apparently healthy children from 9 kindergarten schools were inoculated on L20B, RD and Hep-2C cell lines. A total of 66 (24.2%) isolates were obtained with 28 (42%) from males. There was a higher isolation rates (56%) among younger children aged two and below than in older children between the ages of 3 years and above. Of these, 31 (11.4%) and 12 (4.4%) respectively showed growth on Hep-2C and RD cells only while 23 (8.4%) showed growth on both cell lines. There was no isolation on L20B cell line. Most (25.8%) of the isolates were obtained from BD school with the least (1.5%) from KM school as shown in Figure 1. Substantial amount of isolates were also obtained from MS, MU and TS schools. As shown in Table 1, majority 47% of the isolates were harvested on Hep-2C only compared with RD cell line while 34.8% were collected on both RD and Hep-2C. The results also indicated that isolates were made on Hep-2C from all the 9 schools while isolates on RD cells were harvested from 6 schools (Figure 2).

**Table 1:** Summary of isolates identified on different cell lines

	RD	Hep-2C	RD + Hep-2C
Cox B	4	3	3
Echo 2	0	0	1
Echo 4	1	0	2
Echo 6	0	1	0
Echo 7	2	1	1
Echo 11	1	0	0
Echo 13	1	4	0
Echo 14	0	1	0
Echo 20	0	2	0
Echo 25	0	1	0
Echo 29	0	1	0
Echo 27	0	0	1
Echo 33	0	1	0
Untypable	3	16	15
**Total**	12	31	23

All isolates were selected for antigenic characterization using standard polyclonal sera from the National Institute of Public Health and Environment (RIVM), Bilthoven, The Netherlands. A total of 32 (48.5%) isolates were identified by the antisera pools. All viruses belonged to the human enterovirus species B. Of the identified NPEV, 13 different serotypes were characterized as shown in Table 1. Echovirus 6, Echovirus 14, Echovirus 25, Echovirus 29 and Echovirus 33 grew on Hep-2c cells only while Echovirus 11 grew on RD cells only. Echovirus 27 was however seen to grow on both RD and Hep-2C cell lines. Of the Coxsackie B virus that grew, 4 showed growth on RD only, 3 on Hep 2-C only and 3 grew on both cell lines. A total of 10 (15%) children excreted Coxsackie B viruses with the highest prevalence from TC school followed by AME and BD schools. Echovirus 13 was detected in 3 schools with MU school being predominant. However, there was no significant difference in the distribution of serotypes (p< 0.001 Fisher's exact test) in the schools. Table 2 shows the distribution of virus by age group. Children in the 2-5 years age group excreted more (25.3%) virus than the other groups but the difference is statistically not significant (P = 0.001).

**Table 2:** Non-polio enterovirus positive samples by age of school children

Age	Samples tested	No of Isolates (%)	Coxsackie B virus	Echovirus	Untypable
0-2	16	4 (25%)	0	2	2
3-5	198	50 (25.3%)	10	16	24
>6	59	12 (20.3%)	0	4	8
**Total**	**273**	**66 (24.2%)**	**10**	**22**	**34**

**Figure 3:**
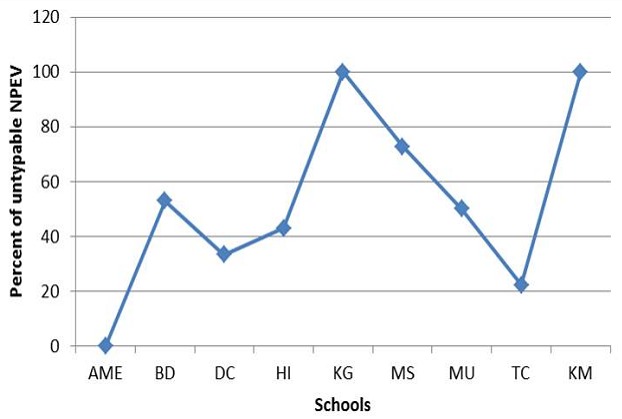
Frequency of untypable NPEVs in the nursery schools

The A-G and Coxsackie B pool anti sera used for the microneutralisation assay could not identify 34 (51.5%) of the isolates. Sixteen (47.1%) of these isolates grew on Hep-2C only, 3 (4.5%) on RD only and 15 (44.1%) on both cell lines. The distribution of the untypable NPEVs is shown in Figure 3. All the isolates from KG and KM schools were untypable.

## Discussion

Virus isolation and serotyping by virus neutralization assay which allow the identification of NPEV serotypes [15] (Muir et al, 1998) are the World Health Organization standard protocol for enterovirus surveillance. The study employed this standard protocol to survey the prevalence of enterovirus circulation among apparently healthy school children in a sub-metropolitan assembly. NPEVs are common in children; however, its importance in the Ghanaian school children has not been well documented.

A total of 66 out of 273 samples tested found to grow on the cells given a NPEV rate of 24.2%. Similar results were obtained in Egypt and Italy where NPEV rate ranged from 17.6% to 65% with no wild polio isolation from 1000 and 1551 healthy children respectively [16, 17]. The high rate in this study was made possible by the combination of two cell lines RD and Hep-2C. The use of RD cells only produced a rate of 13% while Hep-2C gave 19.8% isolation rate. This finding further confirms that the withdrawal of Hep-2C cell line from the NPEV isolation protocol reduces virus isolation rate (WHO, 2004). This is also true because in acute flaccid paralysis surveillance, poliovirus is the pathogen of interest. Gender distribution of NPEV in males 38/143 (26.6%) was found to be higher than in females 38/130 (21.5%) which conforms to a recent finding in India [18]. This is contrary to a large body of data observation that enteroviral diseases, and possibly also enteroviral infections, occur more frequently in males than in females [13, 19, 20]. They suggest that biological reasons are responsible for the higher infection rate among males such as longer duration of virus excretion and higher virus titre in stools of males. It has been found that age is one of the most important determinants of enteroviral infection outcomes, with different age groups having different susceptibilities to infection [21]. In this study, the isolation rate among the different age groups was similar with the age 2-5 years’ group having a slight edge over the rest. This finding however contradicts the findings of work done in India where the isolation rate was significantly higher in the age 0-2 years’ group [18].

The limitation of this study was the use of the polyclonal antisera pools which only identifies a limited number of enteroviruses rather than molecular typing which identifies all the circulating serotypes. Secondly, the total oral polio vaccine history of the school children was unknown. About 48.5%8 (32/66) of the NPEV isolates were not identified by neutralization test which is similar to findings in Netherlands, India and Egypt [22, 23]. This could possibly be due to “breakthrough” because of high-viral concentration used in neutralization test rendering an inconclusive pattern or antigenic drift [18]. Other viruses including reoviruses and adenoviruses could also not be ruled out [24]. During the serotyping, all wells containing polio pool antisera did not neutralize implying that these isolates are NPEVs and not polioviruses. The serotyping identified 66/273 (24.2%) isolates comprising 13 different serotypes with Coxsackie B viruses being predominant followed by Echovirus 13 and Echovirus 7. These same NPEVs were found predominant in 2009 during NPEV survey from AFP cases [25] and could be endemic in the country [26]. Together, the echoviruses were found to be more than the Coxsackie viruses. This results correlates with a study conducted by Kuramitsu and colleagues where the transmissibility of echovirus was found to be high, while Coxsackie A virus infection was self-limiting [20]. In an epidemiological survey of non-polio enterovirus among families in Mongolia conducted in the late summer of 2003, where stools of 122 healthy persons were collected weekly for 5 weeks, eight serotypes of non-polio enteroviruses (echovirus 30, 33, 12, 25, coxsackievirus) were isolated from 62 persons [20]. Enteroviruses such as Coxsackie and echoviruses, are among the most common and significant causes of infectious illness in infants and children [27]. Consistent with our findings, studies involving serotyping of NPEV isolated from stool specimens among healthy children in India and Egypt found Echovirus 6, 11, 9 and Coxsackie virus B to be prevalent [16, 28]. NPEVs differences observed in different geographical areas could be that within even a given geographic locality, some serotypes may be endemic, with little or only gradual change in the range of serotypes present from year to year. In temperate climates there is increased circulation in summer and early fall. In contrast, other serotypes maybe introduced periodically, causing epidemics, with very few isolations reported in intervening years [10].

Our study further suggests that NPEVs are in circulation in all the school with the majority in BD school. Hygienic conditions surrounding the schools may be a contributing factor. The school children have to be educated on personal hygiene of simply washing of hands before eating and also ensure that they do not defecate indiscriminately.

## Conclusion

This study was able to detect thirteen different non-polio enterovirus serotypes among apparently healthy school children. The rate of isolation was 24.2 with the highest prevalence from BD school. No poliovirus was isolated from the study. The high rate of untypables NPEV found call for the need for molecular typing to allow the detection of the full range of serotypes in circulation among the children since these HEV's are of importance in relation to the fatal diseases they cause and should be extensively investigated among apparently healthy children.

## References

[1] Pallansch MA, Roos RP, Knipe DM, Howley PM, Griffin DE, Lamb RA, Martin MA, Roizman B, Straus SE (2001). Enteroviruses: polioviruses, coxsackieviruses, echoviruses, and newer enteroviruses. Fields virology.

[2] ICTV (2009). Virus Taxonomy: 2009 release. http://www.ictvonline.org/virusTaxonomy.asp?msl_id=25.

[3] Oberste MS, Maher K, Flemister MR, Marchetti G, Kilpatrick DR, Pallansch MA (2000). Comparison of classic and molecular approaches for the identification of untypeable enteroviruses. J Clin Microbiol.

[4] Oberste MS, Nix WA, Maher K, Pallansch MA (2003). Improved molecular identification of enteroviruses by RTPCR and amplicon sequencing. J Clin Virol.

[5] Chan LG, Parashar UD, Lye MS, Ong FGL, Zaki SR (2000). Deaths of childrenduring an outbreak of hand, foot, and mouth diseasein Sarawak, Malaysia: clinical and pathoilogicalcharacteristics of the disease. Clin Infect Dis..

[6] Theoklis Z, Klein DJ (1998). Enterovirus infection. Pediatr Rev..

[7] Kearney MT, Cotton JM, Richardson PJ, Shah AM (2001). Viral myocarditis and dilated cardiomyopathy: mechanisms, manifestations, and management. Postgrad Med J..

[8] Hughes SA, Thaker HM, Racaniello VR (2003). Transgenic mouse model for Echovirus myocarditis and paralysis. Proc Natl Acad Sci USA..

[9] World Health Organization (2013). Tracking progress towards global polio eradication, 2011-2012. Weekly Epidemiol Record.

[10] Morens DM (1978). Enteroviral disease in early infancy. J Pediatr..

[11] Grassly NC, Jafari H, Bahl S, Durrani S (2010). Asymptomatic wild-type poliovirus infection in India among children with previous oral poliovirus vaccination. J Infect Dis..

[12] Odoom JK, Forrest L, Dunn G, Osei-Kwasi M, Obodai E (2012). Interruption of poliovirus transmission in Ghana: molecular epidemiology of wild-type 1 poliovirus isolated from 1995-2008. J Infec Dis..

[13] Baba MM, Oderinde BS, Patrick PZ, Jarmai MM (2012). Sabin and Wild Polioviruses from Apparently Healthy Primary School Children in Northeastern Nigeria. J Med Virol..

[14] WHO (2004). World Health Organization: PolioLaboratory Manual. Department of Vaccines and Biologicals.

[15] Odoom JK, Obodai E, Barnor JS, Ashun M, Arthur-Quarm J, Osei-Kwasi M (2012). Human Enteroviruses isolated during acute flaccid paralysis surveillance in Ghana: implications for the post eradication era. The Pan African Med J..

[16] Muir P, Kammerer U, Korn K, Mulders MN (1998). Molecular typing ofenteroviruses: current status and future requirements. Clin Microbiol Rev..

[17] Afifi SA, Samar HZ, Aly FF, Hend EH (2009). Isolation and Identification of Non-Polio: Enteroviruses from Children in Different Egyptian Governorates. Aus J of Basic and Applied Sci..

[18] Rifqiyah NU, Dhenni R, Jajuli A, Nishimura Y, Shimizu H, Utama A (2009). Detection and Identification of Human Enteroviruses among Healthy Children in Antajaya, Bogor. J Biotechnology Res in Tropical Region.

[19] Laxmivandana R, Yergolkar P, Gopalkrishna V, Chitambar SD (2013). Characterization of the Non-Polio Enterovirus Infections Associated with Acute Flaccid Paralysis in South-Western India. PLOS ONE.

[20] Brown EH (1992). Enterovirus infection. Br Med J..

[21] Kuramitsu M, Kuroiwa C, Yoshida H, Miyoshi M, Okumura J, Shimizu H, Narantuya L, Bat-Ochir D (2005). Non-polio enterovirus isolation among families in Ulaanbaatar and Tov province, Mongolia: prevalence, intrafamilial spread, and risk factors for infection. Epidemiol Infect..

[22] Kargar M, Sadeghipour S, Nategh R (2009). Environmental surveillance of non-polio enterovirus in Iran. Virol J..

[23] Verboon MA, Krediet TG, Van AM, Loon J, Kaan JMD, Galama LG, Gerards Fleer A (2002). Epidemiological survey of neonatal non-polio enterovirus infection in the Netherlands. J Med Virol..

[24] Dhole TN, Ayyagari A, Chowdhary R, Shakya AK (2009). Non-polio enteroviruses in acute flaccid paralysis children of India: vital assessment before polio eradication. J PaediatrChild Health..

[25] Hughes MS, Hoey EM, Coyle PV (1993). A nucleotide sequence comparison of coxsackievirus B4 isolates from aquatic samples and clinical specimens. Epidemiol Infect..

[26] Kapoor A, Ayyagari A, Dhole TN (2001). Non-polio enteroviruses in acute flaccid paralysis. Indian J Pediatr..

[27] Apostol LN, Suzuki A, Bautista A, Galang H, Paladin FJ (2012). Detection of Non-Polio Enteroviruses From 17 Years of Virological Surveillance of Acute Flaccid Paralysis in the Philippines. J Med Virolo..

[28] Grist NR, Bell EJ (1984). Paralytic poliomyelitis and non-polio enteroviruses: studies in Scotland. Rev Infect Dis..

